# ZNF384‐Driven Fibulin‐1 Exacerbates Vascular Stiffness via TGF‐β/Smad3‐Mediated Senescence and Fibrosis

**DOI:** 10.1096/fj.202501262RR

**Published:** 2026-03-12

**Authors:** Dan Yan, Tianyi ji, Xiaolu Liang, Mandi Luo, Yi Huang, Pengcheng Luo, Zhen Yang, Le Zhang, Tao Li, Yong Ping Bai, Cuntai Zhang, Lei Ruan

**Affiliations:** ^1^ Department of Geriatrics, Tongji Hospital, Tongji Medical College Huazhong University of Science and Technology Wuhan China; ^2^ China National GeneBank Shenzhen Guangdong China; ^3^ Department of Geriatrics Medicine. Center of Coronary Circulation, Xiangya Hospital Central South University Changsha China

**Keywords:** cell senescence, Fibulin‐1, TGF‐β, vascular stiffness, ZNF384

## Abstract

Vascular stiffness, a hallmark of aging and cardiovascular disease, involves vascular smooth muscle cell (VSMC) senescence and extracellular matrix (ECM) dysregulation. This study investigates the role of fibulin‐1 (Fbln1) in these processes. Plasma proteomic profiling identified dysregulated proteins in vascular stiffness pedigrees. Fbln1 knockout mice and dual vascular stiffness models (natural aging and chronic angiotensin II [Ang II] infusion) were established. Phenotypic assessments included pulse wave velocity (PWV), histology, and molecular markers. Mechanistic investigations integrating DNA pull‐down assays, dual‐luciferase reporter assays, and RNA sequencing (RNA‐seq) were employed to dissect the transcriptional and signaling axis regulating Fbln1 expression and function. Elevated plasma Fbln1 correlated with hereditary vascular stiffness. Both aging and Ang II promote vascular stiffness, whereas Fbln1 knockdown ameliorates this phenotype by reducing PWV, reversing VSMC senescence, and attenuating collagen deposition. Zinc Finger Protein 384 (ZNF384) was identified as a transcriptional activator of Fbln1, which promoted VSMC senescence and collagen deposition via transforming growth factor‐beta (TGF‐β)/SMAD family member 3 (Smad3). Inhibiting TGF‐β/Smad3 signaling abolished Fbln1‐driven senescence and ECM remodeling. Fbln1 exacerbates vascular stiffness through ZNF384‐mediated transcriptional activation and TGF‐β/Smad3‐dependent ECM/senescence pathways. Targeting Fbln1 or its regulators may offer therapeutic strategies for age‐related vascular pathologies.

## Graphical abstract

This article elucidates the regulatory molecular mechanisms of Fibulin‐1 (Fbln1) in vascular stiffness and explores potential therapeutic interventions. Zinc Finger Protein 384 (ZNF384) binds to the Fbln1 promoter, driving its expression. Fbln1 activates the TGF‐β/Smad3 axis, promoting vascular smooth muscle cell (VSMC) senescence and collagen deposition, leading to vascular stiffness.

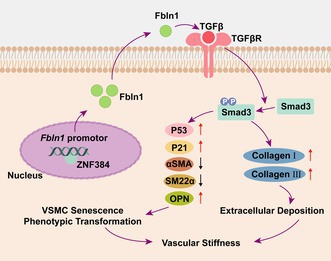

**Abbreviations**
AngII: Angiotensin II
ECM: extracellular matrix
ECM: extracellular matrix protein
EVG: Elastica van Gieson
Fbln1: Fibulin‐1
H&E: hematoxylin and eosin
OPN: osteopontin
p‐Smad3: phosphorylated SMAD family member 3
PWV: pulse wave velocity
RNA‐seq: RNA sequencing
SM22α: smooth muscle protein 22α
Smad3: SMAD family member 3
SNP: Sodium nitroprusside
TGF‐β: transforming growth factor‐beta
VSMCs: vascular smooth muscle cells
Zinc Finger Protein 384: ZNF384
αSMA: α‐smooth muscle actin

## 1 Introduction

Vascular stiffness serves as a pivotal pathological driver for the development of diverse severe cardiovascular diseases and represents a shared etiological mechanism underlying multiple chronic diseases in the elderly population [1, 2, 3]. Currently, there is a lack of effective early diagnostic modalities for vascular stiffness, and therapeutic interventions primarily focus only on mitigating risk factors [4]. Early detection and intervention of vascular stiffness are pivotal measures for assessing and preventing vascular stiffness and its associated vascular disorders. Elucidating the precise molecular mechanisms and molecular targets involved in vascular stiffness and leveraging this knowledge to develop efficacious drugs for early intervention and deceleration of vascular stiffness holds paramount medical significance and societal relevance.

Fibulin‐1 (Fbln1) is an extracellular matrix protein (ECM) that is ubiquitously expressed throughout the arterial wall, with higher expression observed in the medial layer and the region adjacent to the adventitia [5]. Studies have revealed age‐related alterations in Fbln1 levels within the aortic wall. In individuals with diabetes, Fbln1 accumulates within the arterial wall, serving as a marker for EC remodeling and a hallmark of arterial sclerosis. Furthermore, increased plasma concentrations of Fbln1 in diabetic patients have been associated with augmented overall mortality and cardiovascular mortality rates [6]. Enhanced expression of Fbln1 has also been observed in individuals with atherosclerosis [7], suggesting its involvement in the pathophysiological processes underlying vascular aging‐related cardiovascular disorders, often concomitant with heightened vascular rigidity. However, the molecular mechanisms governing Fbln1 in vascular stiffening, from the upstream signals that regulate its expression to the downstream pathways it modulates, remain elusive and poorly characterized.

Zinc Finger Protein 384 (ZNF384), a C2H2‐type zinc finger protein, functions as a transcription factor and plays a critical role in modulating cell adhesion, proliferation, and migration processes [8]. Furthermore, ZNF384 binds to the promoter regions of extracellular matrix‐related genes (e.g., the MMP family), modulating ECM remodeling processes [9, 10]. However, whether ZNF384 participates in the regulation of vascular stiffness remains unclear.

Transforming growth factor‐beta (TGF‐β) is widely distributed in the human body [11], with multiple cell types capable of producing TGF‐β and expressing its receptors on their cellular membranes [12]. As a signaling protein, TGF‐β is essential in regulating various cellular functions, including cell proliferation, differentiation, migration, apoptosis, and aging [13]. It exerts crucial regulatory effects on vascular development and normal vascular structure maintenance [14]. TGF‐β binds to its cellular membrane receptor, TGF‐β receptor 2, activating downstream signaling molecule Smad3 through phosphorylation. Phosphorylated SMAD family member 3 (p‐Smad3) translocates into the nucleus and acts as a transcription factor, participating in modulating the expression of target genes [15]. The TGF‐β/Smad3 signaling pathway, as a canonical TGF‐β signaling pathway, is involved in the regulation of vascular smooth muscle cell phenotype transition and plays a significant role in vascular remodeling and atherosclerosis [16]. Whether Fbln1 regulates vascular stiffness through the canonical TGF‐β signaling pathway requires further investigation.

This study aims to identify differentially expressed ECM proteins in familial cohorts with vascular stiffness and elucidate the key molecular mechanisms underlying vascular stiffness.

## 2 Experimental Methods

### 2.1 Human Samples

Two case–control investigations utilizing human biological specimens were conducted. The study protocol received approval from the Ethics Committee of Tongji Hospital, Huazhong University of Science and Technology (Ethics Committee approval number: S248‐1), adhering to the Declaration of Helsinki guidelines. Written informed consent was secured from all participants prior to sample acquisition.

A familial cluster with pathologically increased arterial stiffness was identified through routine cardiovascular screening at Tongji Hospital. Affected individuals manifested accelerated arteriosclerosis, as confirmed by elevated pulse wave velocity (PWV ≥ 1400 cm/s), while unaffected family members served as internal controls (PWV within age‐adjusted normal ranges). Inclusion criteria: (1) age ≥ 18 years; (2) availability of detailed medical history; (3) absence of secondary causes of arteriosclerosis (e.g., chronic kidney disease, diabetes mellitus). Exclusion criteria: (1) acute cardiovascular events within the preceding 6 months; (2) use of vasoactive medications (e.g., nitrates, calcium channel blockers). Peripheral blood samples were collected under fasting conditions. The basic information of patients is shown in the (Table S1).

Vascular tissues from elderly patients (*n* = 5) were collected from the cardiovascular tissue repository under the Department of Cardiovascular Surgery at Tongji Hospital (2019–2021). Vascular tissues from young donors were obtained during organ procurement procedures for heart transplantation. The basic information of patients is shown in the (Table S2).

### 2.2 Mice and Experimental Models

All animal experiments were conducted in accordance with the guidelines of the National Institutes of Health and approved by the Experimental Animal Research Committee of Tongji Medical College, Huazhong University of Science and Technology. The ethical approval number for this experiment is TJH‐202104016. Mice were housed in specific pathogen‐free conditions. To generate inducible systemic Fbln1 knockout mice, Fbln1^flox/flox^ homozygous mice are crossed with CAG‐Cre ERT2 transgenic mice expressing tamoxifen‐inducible Cre recombinase under the ubiquitous CAG promoter. Offspring with the genotype Fbln1^flox/flox^×CAG‐Cre ERT2 (Fbln1^−/−^) are obtained. Control groups consisted of Cre‐negative littermates designated as WT (wild type). Due to the embryonic lethality associated with constitutive Fbln1 deletion, tamoxifen (100 mg/kg/day, administered intraperitoneally for 7 consecutive days) is delivered postnatally at 4 weeks of age to activate Cre‐ERT2, thereby inducing recombination and excision of the floxed Fbln1 allele. We employed the classic Angiotensin II (AngII)‐induced vascular aging model, in which male 6–8 week‐old WT or Fbln1^−/−^ mice were continuously infused with AngII at 8 weeks of age using subcutaneously implanted osmotic minipumps (model 2004, Alzet, CA, USA). After subcutaneous implantation in the back of the mice, AngII powder (Sigma) was dissolved in sterile saline and loaded into the osmotic minipumps for systemic hormone delivery (infusion rate of 1000 ng/kg/min, lasting for 28 days). Control group mice were gavaged with sterile saline. After 28 days, the mice were assessed for relevant aging phenotypes.

### 2.3 Mouse Pulse Wave Velocity (PWV) Measurement

Mouse PWV was assessed using a Doppler ultrasound system (Mouse Doppler, Indus Instruments). Following anesthesia with isoflurane, mice were positioned in a supine position on a heated platform maintained at a constant temperature of 38°C. The limbs of the mice were immobilized on electrocardiogram (ECG) electrode pads, and ECG gel was applied to the distal ends of the limbs. Continuous ECG recordings were obtained. Pressure waveforms were detected at the descending aorta and abdominal aorta using a 20 MHz probe. The arrival and transit times of five consecutive cardiac cycles were calculated, and the distance between the descending aorta and abdominal aorta was measured using a calibrated ruler. Mouse PWV (m/s) was determined by dividing the propagation distance by the corresponding propagation time.

### 2.4 Mouse Blood Pressure (BP) Measurement

Using the Coda 4.2 (Kent Scientific, USA) non‐invasive blood pressure monitor, we measured the blood pressure in mice. Secure the mouse to the heating pad and ensure the surrounding environment is quiet. Place the mouse's tail in the inflation‐deflation holder and connect it to the blood pressure monitor for measurement. Each mouse underwent 15 measurements while simultaneously recording the heart rate.

### 2.5 Detection of Reactive Oxygen Species (ROS)

The CM‐H2DCFDA dye solution (Wuhan, Beyotime Biotechnology, S0033) was prepared by diluting it 1:1000 with PBS. An appropriate volume of the diluted DHE dye solution was added to completely cover the blood vessel sections. The sections were incubated at 37°C for 30 min. During incubation, the samples were gently inverted every 3–5 min to ensure thorough contact between the probe and the cells. The sections were then washed three times with PBS to remove any CM‐H2DCFDA that had not entered the cells. Finally, the results were observed and analyzed under a fluorescence microscope.

### 2.6 Cell Culture

Primary vascular smooth muscle cells (VSMCs) were isolated from the aortas of C57BL/6 mice. The vascular adventitia was first dissected, followed by mincing the vessels into small fragments. Enzymatic digestion was then performed to disaggregate the tissue into single cells. VSMCs were cultured in a specialized medium composed of F12 medium (high‐glucose), 10% fetal bovine serum, and 1% penicillin–streptomycin solution. Cells were maintained in gelatin‐coated culture flasks and incubated at 37°C in a humidified 5% CO2 atmosphere. The culture medium was replaced every 2–3 days, and cells were subcultured at 80%–90% confluence.

### 2.7 Vascular Tension Measurement

The fresh mouse aorta was isolated and mounted in a tissue chamber (MT 620 M, Denmark). The chamber was filled with oxygenated Physiological Saline Solution (PSS) buffer. The tissue was connected to a force transducer, followed by oxygenation and heating, and allowed to equilibrate for 40–60 min. The data recorder was then started for continuous recording. The vessel was stretched to an initial pre‐tension of approximately 3–5 mN. The PSS in the bath was drained and promptly replaced with oxygenated High potassiμm Physiological Saline Solution (KPSS), which was applied for 10 min. Subsequently, phenylephrine was administered to induce aortic contraction. After a 10 min stabilization period, vasodilator stimulation was initiated. Sodium nitroprusside (SNP) was added cumulatively from low to high concentrations, each concentration being applied for 30 s while data were recorded.

### 2.8 Histology and Immunohistochemistry

Mouse and human arteries were fixed in 4% paraformaldehyde and embedded in paraffin. Horizontal‐sectional slices of 4 μm thickness were prepared for morphological evaluation using hematoxylin and eosin (H&E) staining as well as Elastica van Gieson (EVG) and Masson staining. Vascular structure was analyzed under electron microscopy. Image J software was utilized for further image analysis.

### 2.9 Senescence‐Associated β‐Galactosidase (SA‐β‐Gal) Staining Assay

SA‐β‐gal staining experiments were performed using the Bluo‐Gal β‐Galactosidase Staining Kit. Primary vascular smooth muscle cells (VSMCs) were seeded in a 24‐well plate and allowed to adhere. Afterward, cells were treated according to the specific experimental protocols. Following cell treatment, the culture medium was aspirated, and cells were washed once with PBS. Then, 200 μL of fixative solution was added to each well and incubated at room temperature for 20 min. The fixative solution was removed, and cells were washed three times with PBS. The β‐Galactosidase staining solution was prepared by mixing 930 μL of solution C, 10 μL of solution A, 10 μL of solution B, and 50 μL of X‐gal staining solution. Next, 250 μL of the staining solution was added to each well and incubated at 37°C overnight. The following day, the staining solution was aspirated, and cells were washed three times with PBS to remove any residual staining solution. Images were captured under a microscope for observation, counting, and analysis of the results.

### 2.10 Immunofluorescence (IF) Staining

VSMCs were planted in a 6‐well plate. After cell adhesion, cells were stimulated with AngII for 72 h. Following cell stimulation, cells were washed and fixed with 4% paraformaldehyde at room temperature for 15 min. After two washes with PBS, cells were permeabilized with PBS buffer containing 0.3% Triton X‐100 and 5% normal goat serum at room temperature for 1 h. Subsequently, cells were incubated overnight at 4°C with a rabbit polyclonal antibody against p‐Smad3 (diluted 1:50). After overnight incubation, cells were washed three times with PBS buffer containing 0.1% Tween‐20. Then, cells were stained with Cy3‐conjugated anti‐rabbit secondary antibody (diluted 1:100; Jackson ImmunoResearch Laboratories, West Grove, PA) at room temperature for 1 h, followed by nuclear staining with DAPI (Vector Laboratories, Burlingame, CA, USA) for 5 min. Digital images were captured using a fluorescence microscope.

### 2.11 Quantitative Polymerase Chain Reaction (q‐PCR)

Total RNA was extracted using the TRIzol method (Invitrogen) and quantified with a NanoDrop spectrophotometer. Reverse transcription was carried out with the PrimeScript RT Reagent Kit (Takara) following the manufacturer's instructions. The preparation of the qPCR reaction mixture is as follows: template (cDNA), primers, and nuclease‐free water and enzyme mix. The amplification was performed on a QuantStudio 6 Flex system using a specified thermal profile. The *Fbln1* gene was amplified using specific primers (forward: 5′‐GATGGAAATCAGATGGCTAACC‐3′; reverse: 5′‐CCAGTTGGTTGTGACAACATTG‐3′). GAPDH was used as the endogenous control, and relative gene expression was determined using the 2^−ΔΔCt^ method, with three technical replicates for each sample. Data were included only if they met the established quality thresholds.

### 2.12 Western Blot Analysis

The cultured cells were collected and lysed in RIPA buffer containing protease inhibitors. The supernatant from each sample was collected and total protein concentration measured using the BCA protein assay kit. Equal amounts of the protein lysates were resolved in SDS‐PAGE and transferred to a PVDF membrane. Then the membranes were incubated with the following primary antibodies at 4°C overnight: P53 (sc‐6246, Santa Cruz, 1:1000), P21 (R&D, MAB1355, 1:800), Fbln1 (sc‐374 539, Santa Cruz, 1:1000), osteopontin (OPN, sc‐73 631, Santa Cruz, 1;800), smooth muscle protein 22α (SM22α, 10 493–1‐AP, Proteintech, 1:1000), α‐smooth muscle actin (αSMA, #19245, CST, 1:1000), Collagen I (ab270993, Abcam, 1:1000), Collagen III (ab184993, Abcam, 1:1000), TGF‐β (#3709, CST, 1:1000), Samd3 (#9523, CST, 1:1000), P‐Samd3 (ab52903, Abcam, 1:1000) or GAPDH (#2118, CST, 1:5000). After washing with TBST, the blots were developed with secondary antibodies conjugated to horseradish‐peroxidase and visualized by enhanced chemiluminescence detection kit.

### 2.13 DNA Pull‐Down

Construct a PUC57 plasmid containing the biotinylated Fbln1 promoter. Amplify and purify the Fbln1 DNA probe, and verify that its size is approximately 2000 bp. Mix Nucleic Acid‐Compatible Streptavidin Magnetic Beads with the Fbln1 DNA probe, and add an equal amount of non‐biotinylated DNA to the control tube. Adjust to equal volumes using Nucleic Dilution Buffer and incubate at room temperature for 2 h with a vertical rotator. Place the tube on a magnetic stand to allow bead separation, and carefully discard the supernatant. Repeat this process three times. Lyse the cells on ice using cell lysis buffer for 30 min, vortexing every 5 min. Centrifuge at 12000 rpm for 20 min at 4°C, and collect the protein supernatant. Add 400 μL of the extracted protein to both the control and experimental tubes, and incubate overnight at 4°C using a rotator. Finally, perform magnetic separation to isolate the protein‐bead complexes. Add 100 μL of Elution Buffer to elute the proteins, mix thoroughly, and heat in a boiling water bath for 9 min. Place the tube on a magnetic stand for 2 min, and collect the protein supernatant for subsequent mass spectrometry analysis.

### 2.14 Protein Mass Spectrometry

For proteomic identification by mass spectrometry of plasma or IP samples, lipids and carbohydrates must first be depleted to prevent analytical interference. This can be accomplished using separation techniques such as gel electrophoresis or high‐performance liquid chromatography. The Orbitrap Fusion Lumos mass spectrometer (Thermo Fisher Scientific) coupled with an EASY‐nanoLC 1200 system was used. A 3 μL sample was injected and separated over a 60 min gradient (300 nL/min, 55°C), starting at 4% B, followed by a nonlinear increase to 50% B in 53.6 min, a rapid rise to 95% B in 40 s, and a 5.6 min hold at 95% B. Data‐dependent acquisition (DDA) mode was employed, with automatic switching between MS and MS/MS scans.

### 2.15 Chromatin Immunoprecipitation (ChIP)

The collected smooth muscle cells were mixed with 11% formaldehyde in PBS and rotated at room temperature for 10 min. Glycine Buffer (Biology, Wuhan, BOLG2309) was added to quench the cross‐linking, followed by cell pellet collection. The pellet was lysed on ice with Lysis Buffer for 30 min, then mixed with ChIP Buffer. After sonication at low temperature, the supernatant was collected by centrifugation. RNase A, NaCl, and Proteinase K were added to the supernatant and incubated overnight, after which DNA was recovered. ZNF384 antibody or species‐matched IgG was added to the respective sample groups overnight. Samples were then added to pre‐treated magnetic bead tubes and rotated at 4°C for 2 h. The beads were resuspended with different Wash Buffers, and the final pellet was collected after supernatant removal. Elution Buffer was added to the pellet to purify the DNA again. The samples were used for subsequent sequencing and qPCR.

### 2.16 Dual‐Luciferase Reporter Assay

The Fbln1 promoter sequences (wild‐type and mutant) were cloned into the pGL3‐Basic vector containing the firefly luciferase gene and co‐transfected into HEK‐293 T cells with an internal control plasmid at a ratio of 20:1. After 36 h of culture post‐transfection, the cells were lysed using lysis buffer. A dual‐luciferase reporter assay kit (Promega) was employed, starting with the addition of Luciferase Assay Reagent II to measure firefly luciferase activity, followed by the addition of Stop & Glo Reagent to measure Renilla luciferase activity. The luminescence values were detected using a chemiluminescence microplate reader, and the ratio of firefly to Renilla luciferase activity was calculated to ultimately compare the relative transcriptional activity of Fbln1 between the experimental and control groups.

### 2.17 Data Analysis

Statistical analysis was performed using SPSS 24.0 software. Continuous data were presented as mean ± standard deviation (*x* ± sd). Between‐group comparisons were conducted using *t*‐test and one‐way analysis of variance. Categorical data were presented as percentages and between‐group comparisons were performed using the chi‐square test (*χ* ^2^ test). A two‐tailed *p* < 0.05 was considered statistically significant.

## 3 Results

### 3.1 Elevated Fbln1 Levels in Plasma Proteins of Vascular Stiffness Pedigrees

**FIGURE 1:**
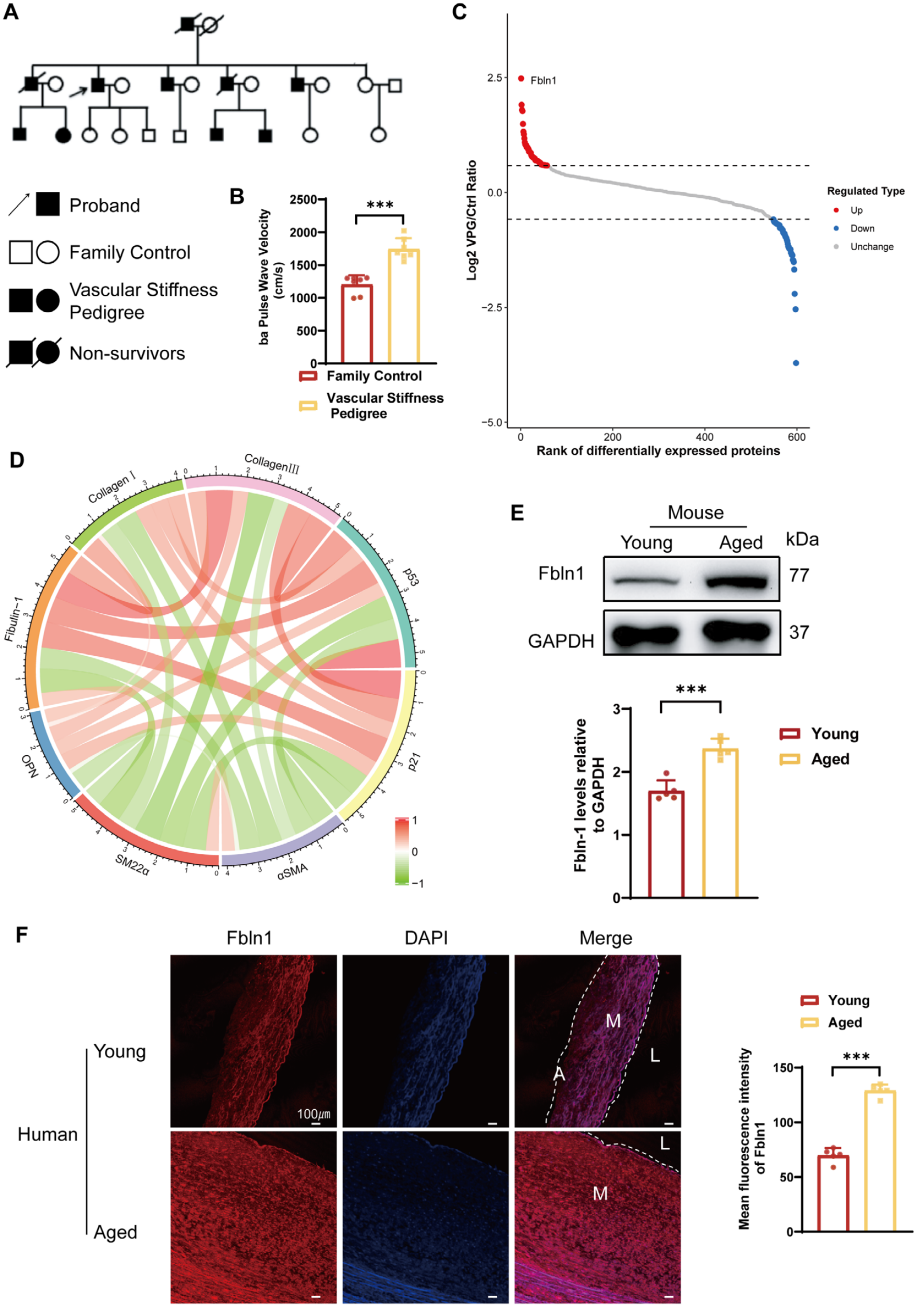
Fibulin‐1 (Fbln1) demonstrates a significant correlation with vascular stiffness. A: Genetic Pedigree of Familial Vascular Stiffness. B: Pulse wave velocity (PWV) in individuals with a familial predisposition to vascular stiffness. C: Differentially expressed plasma proteins between health control and individuals with a familial predisposition to vascular stiffness. D: Co‐expression correlation analysis of genes associated with vascular stiffness and Fbln1 in public aging databases. E: Western blot results showed the expression levels of Fbln1 in vascular tissues of naturally aged mice. F: Immunofluorescence analysis of vascular tissues in young and elderly cohorts. L, lumen; M, tunica media; A, tunica adventitia. *Significant differences. *n* ≥ 5, *p < 0.05, **p < 0.01, ***p < 0.001.

A familial cluster with pathologically increased arterial stiffness was detected during routine cardiovascular assessments in our hospital‐based screening population at Tongji Hospital. Pulse wave velocity (PWV) has been established as the non‐invasive gold‐standard methodology for quantifying arterial stiffness in both clinical and research settings [17]. PWV increases with advancing age [18]. Within this pedigree, multiple individuals exhibited accelerated arteriosclerosis, as illustrated in the genetic pedigree (Figure 1A). Compared to individuals with normal vascular stiffness within the same family, those with vascular sclerosis exhibited a PWV reaching 1800 cm/s (Figure 1B). To investigate the underlying causes of increased vascular stiffness in this family, peripheral blood samples from family members were collected for proteomic analysis. Analysis of differentially expressed proteins revealed a significant elevation of Fbln1 in the plasma of patients with vascular stiffness (Figure 1C). VSMC senescence and ECM deposition drive arteriosclerosis, and senescent smooth VSMCs undergo phenotypic switching from a contractile to a synthetic phenotype [19, 20]. Therefore, we investigated the potential association between Fbln1 and both senescence markers in VSMCs and collagen deposition. We analyzed single‐cell RNA sequencing (scRNA‐seq) data of vascular tissues from young versus aged cynomolgus monkeys ( *Macaca fascicularis* ) in the GEO database. Fbln1 expression exhibited positive correlations with senescence‐associated proteins (p53 and p21) and secretory phenotype markers (OPN), whereas negative correlations were observed with contractile phenotype markers (α‐SMA and SM22α) as shown in Figure 1D. Concomitantly, Fbln1 demonstrated strong positive correlations with collagen I and III expression, suggesting its potential involvement in collagen deposition processes (Figure 1D). Given that Fbln1 is primarily expressed in the media and adventitia of blood vessels, we assessed the changes in VSMCs in naturally aged mice and aged adults. WB analysis showed increased Fbln1 levels in aged mouse vasculature demonstrated upregulated expression of Fbln1 in vascular proteins extracted from naturally aged mice (Figures 1E and S1A–D). Immunofluorescence staining was used to assess the expression levels of Fbln1 in the aorta of young and elderly individuals, revealing that Fbln1 expression is significantly upregulated in the vasculature of aged individuals (Figure 1F). Moreover, we found that this upregulation was particularly evident in elderly vessels covered with atherosclerotic plaques (Figure S1E), a characteristic pathological feature of vascular aging [21].

### 3.2 In Vivo Knockdown of Fbln1 Improved Vascular Stiffness in Aged Mice

Aging contributes to vascular stiffening. We next aim to investigate whether Fbln1 knockout mitigates vascular stiffness in aged mice. The Fbln1 knockdown (Fbln1^−/−^) mouse model was rigorously characterized through tripartite validation: PCR‐based genotyping of the target locus (Figure S1F–G), WB quantification of Fbln1 expression (Figure S1H), and spatially resolved immunohistochemical profiling of Fbln1 distribution in vascular tissue (Figure S1A). We demonstrated that aged Fbln1‐knockout mice, regardless of sex, show no difference in vascular stiffness between males and females, but both exhibit significantly reduced stiffness compared to aged wild‐type controls (Figure S2A). While prior studies indicate that estrogen fluctuations during peri‐menopausal periods affect arterial stiffness in female mice [22], our investigation required controlling for these variables. As estrogen‐mediated effects were not the focus of this study, the experimental cohorts utilized male mice.

**FIGURE 2:**
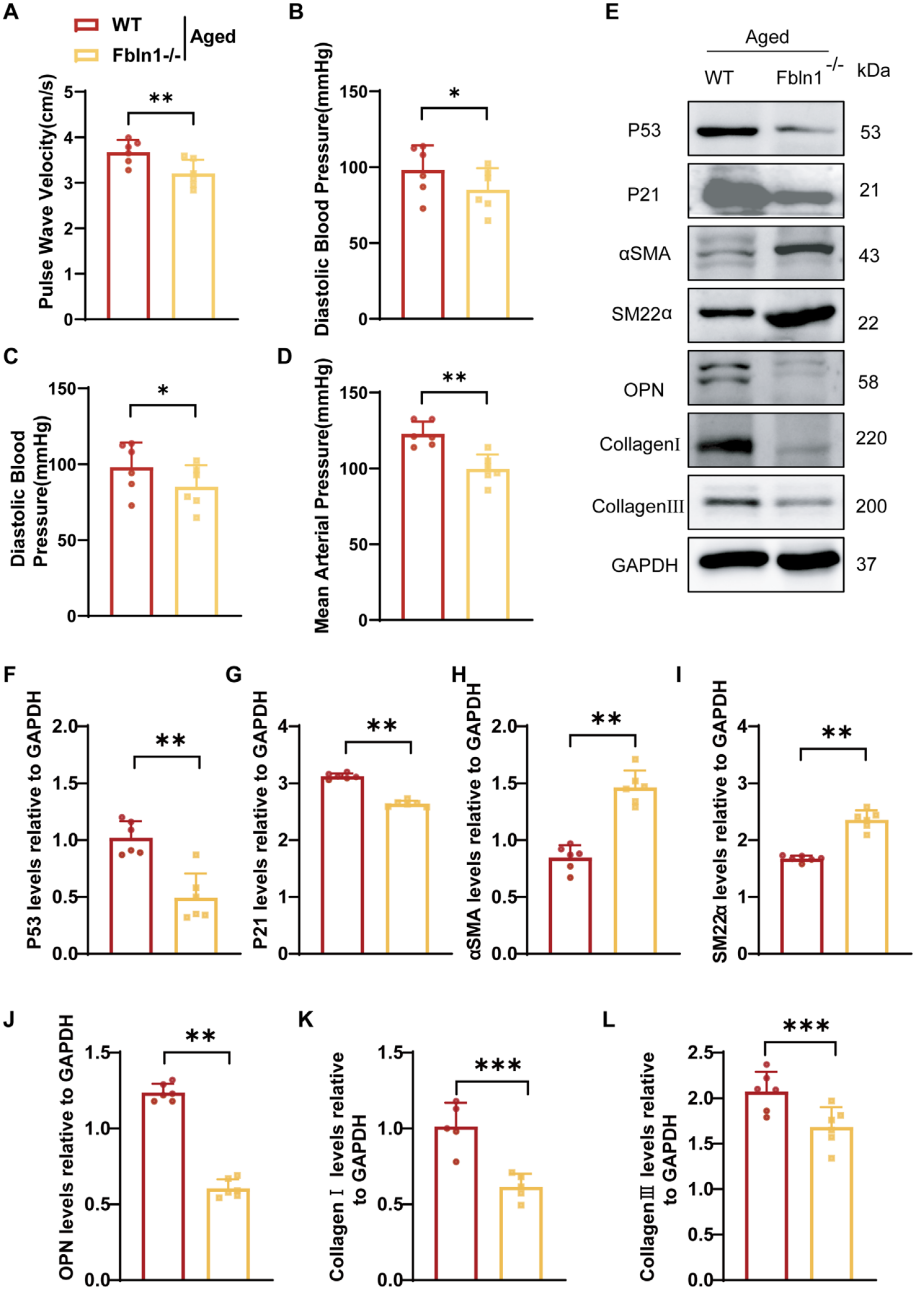
In vivo knockdown of Fbln1 improved vascular stiffness in nature aging mice. A: PWV measurements in aged mice. B–D: Effect of Fbln1 deficiency on systolic blood pressure (B), diastolic blood pressure (C) and mean arterial pressure (D) in normal WT and Fbln1^−/−^ mice is shown. E–L: Western blot results showed that Fbln1 knockdown could modulate the expression of p53, p21, αSMA, SM22α, OPN, collagenI and collagenIII induced by aging in mice. wild‐type mice: WT, Fbln1 knockout mice: Fbln1^−/−^. *Significant differences. *n* ≥ 5, *p < 0.05, **p < 0.01, ***p < 0.001.

Using this approach, we found that knockdown of Fbln1 can ameliorate vascular stiffness in aged mice, as evidenced by a reduction in PWV (Figure 2A) and blood pressure (Figure 2B–D). Additionally, compared to wild‐type (WT) mice, Fbln1 knockout mice exhibited reduced vascular expression of the aging markers p53 and p21 (Figure 2E–G). Fbln1 deficiency reversed cellular phenotypic switching, as evidenced by upregulated α‐SMA and SM22 expression (Figure 2E,H–I), coupled with decreased OPN levels via WB analysis (Figure 2E,J). H&E and Masson staining demonstrated that Fbln1 deficiency significantly attenuates vascular fibrosis, evidenced by reduced collagen deposition (blue/green staining) and preserved vessel wall architecture (Figure S2C,D). Consistent with histopathological staining results, WB analysis confirmed a significant reduction in collagen I and III protein expression within the vasculature of aged mice following Fbln1 knockdown (Figure 2E,K–L). Concurrently, assessment of vasodilatory function revealed that knockdown of Fbln1 improved endothelium‐independent smooth muscle cell relaxation. Following sodium SNP administration, aged mice exhibited impaired smooth muscle relaxation, whereas the Fbln1 knockdown group demonstrated heightened sensitivity to SNP (Figure S2E). Aging significantly elevated ROS levels within the vascular media. Knockdown of Fbln1 effectively reduces this age‐associated ROS accumulation (Figure S2F–G). In summary, genetic ablation of Fbln1 ameliorates vascular stiffness in aged mice through dual mechanisms: attenuation of VSMC senescence pathways and suppression of pathological collagen deposition.

### 3.3 In Vivo Knockdown of Fbln1 Attenuates AngII‐Induced Vascular Stiffness in Mice

**FIGURE 3:**
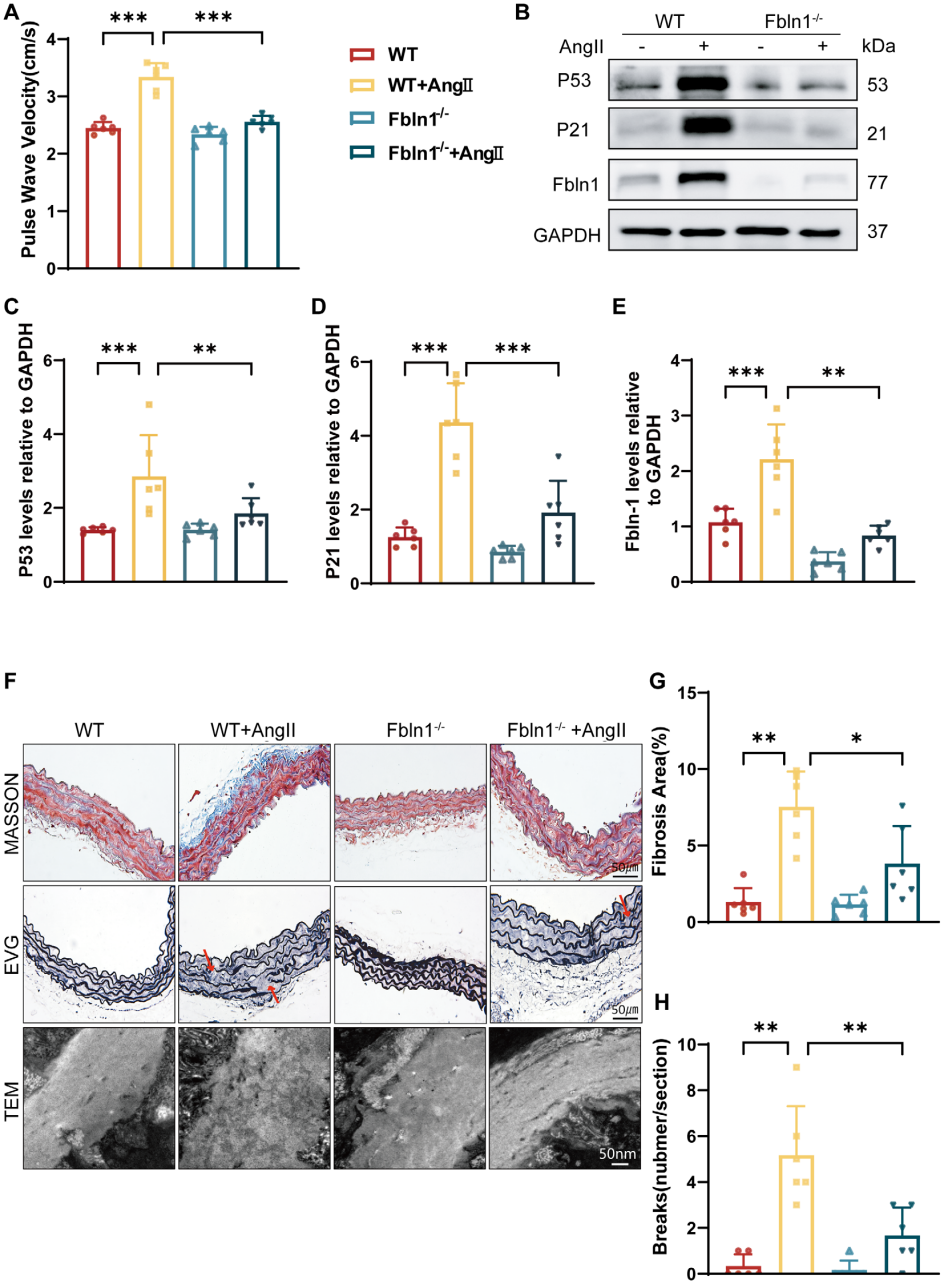
In vivo knockdown of Fbln1 attenuates AngII‐induced vascular stiffness in mice. A: PWV measurements in AngII‐treated and PBS control mice. B–E: Western blot results showed that Fbln1 knockdown could modulate the expression of P53, P21 in mice. F: Assessment of collagen deposition in vascular tissues by Masson's trichrome staining in WT and Fbln1^−/−^ mice (scale = 50 μm). EVG of the vascular wall of the aortic in different groups (scale = 50 μm). The red arrows denote areas of morphological discontinuity in the elastic fibers. Transmission electron microscopy (TEM) of elastin in aortic wall (scale = 50 nm). wild‐type mice: WT, Fbln1 knockout mice: Fbln1^−/−^. *Significant differences. *n* ≥ 5, *p < 0.05, **p < 0.01, ***p < 0.001.

To further investigate whether Fbln1 exacerbates vascular stiffness by modulating aging‐related processes, we established an AngII‐induced vascular stiffness model. Experimental results demonstrated that continuous subcutaneous infusion of AngII significantly increased PWV in mice, while Fbln1^−/−^ mice exhibited a significant improvement in AngII‐induced PWV elevation (Figure 3A). As anticipated, AngII exacerbated VSMC senescence in mice, accompanied by upregulated expression of both p53 and p21 proteins (Figure 3B–E). Comparative analysis with wild‐type (WT) mice revealed significantly attenuated VSMC senescence in Fbln1^−/−^ mice. Concomitantly, Fbln1^−/−^ mice exhibited a marked suppression of pathological phenotypic switching in VSMCs compared to WT controls, as evidenced by WB (Figure S3A–E). Histopathological evaluation via Masson's trichrome and Elastica Van Gieson (EVG) staining demonstrated that AngII administration significantly exacerbated vascular remodeling in WT mice, manifesting as an increase in collagen deposition and an elevation in elastic fiber fragmentation index (Figure 3F). The red arrows indicate the fractured ends of the elastic fibers. Notably, Fbln1 knockdown effectively attenuated these structural aberrations. Ultrastructural analysis by transmission electron microscopy (TEM) revealed that administration of AngII induced pathological disorganization of collagen fibril architecture and caused significant elastic fiber damage within the vascular medial layer. Conversely, knockdown of Fbln1 significantly restored collagen fibril alignment and alleviated the impairment of elastic fibers (Figure 3F). WB analysis further corroborated elevated collagen secretion in the vascular stiffness model, and Fbln1 deficiency markedly attenuated collagen overproduction (Figure S3A,F–G). Fbln1 knockdown attenuated the AngII‐induced accumulation of ROS in VSMCs (Figure S3I), and in the AngII‐mediated model of vascular stiffening, was associated with a marked improvement in VSMC‐dependent vasodilatory function (Figure S3J).

Concurrently, primary VSMCs isolated from mouse aortas were subjected to in vitro Fbln1‐knockdown using siRNA‐mediated gene silencing (Figure S4A–C). Quantification of senescence‐associated β‐galactosidase (SA‐β‐gal) activity revealed that AngII administration elicited a increase in SA‐β‐gal‐positive cells compared to untreated controls, while Fbln1 knockdown significantly attenuated this senescent phenotype (Figure S4D–E). WB analysis further corroborated that Fbln1 knockdown effectively rescued AngII‐induced VSMC senescence (Figure S4F–H), while concomitantly reversing pathological phenotypic switching (Figure S4F–H).

### 3.4 ZNF384 Functions as a Transcriptional Activator That Promotes the Expression of Fbln1

**FIGURE 4:**
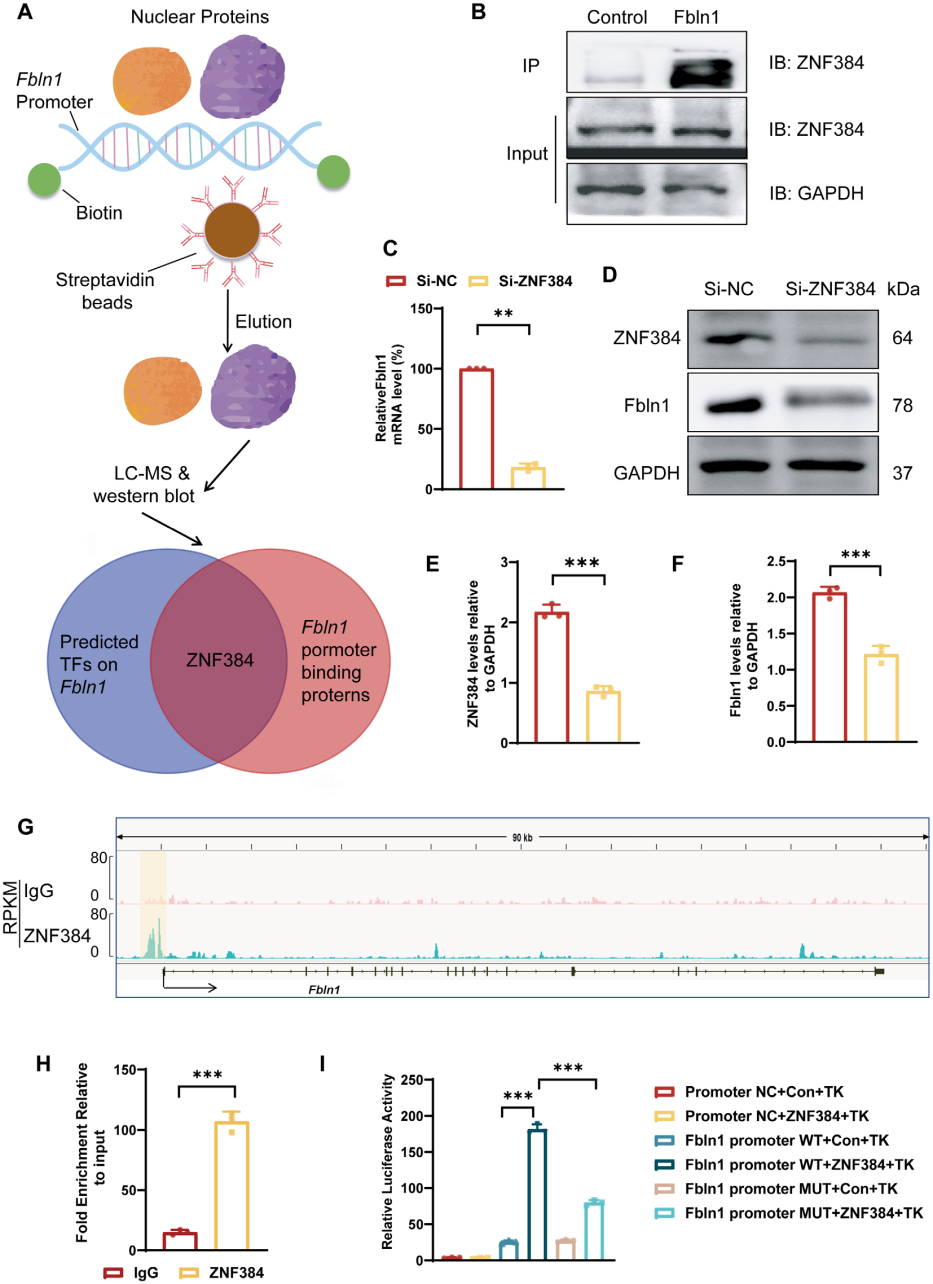
ZNF384 promotes the transcription of *Fbln1*. A: Potential transcription factors regulating Fbln1 were identified through DNA pull‐down assays coupled with protein mass spectrometry. B. Western blot analysis demonstrated direct binding of ZNF384 to the *Fbln1* promoter. C: Knockdown of ZNF384 resulted in decreased *Fbln1* mRNA levels. D–F: Knockdown of ZNF384 led to decreased Fbln1 protein levels. G–H: Both ChIP‐seq and subsequent ChIP‐qPCR experiments confirm the specific binding of ZNF384 to the promoter region of the *Fbln1* gene. I: Dual‐luciferase assay results demonstrated that ZNF384 activates transcription by binding to specific sequences within the *Fbln1* promoter region. *Significant differences. *n* ≥ 3, *p < 0.05, **p < 0.01, ***p < 0.001.

To identify the upstream transcription factors regulating the transcription of the *Fbln1* gene, we combined DNA pull‐down assays with predictive analysis using the UCSC Genome Browser (Figure 4A). The intersection analysis of proteins capable of binding to the *Fbln1* promoter and candidate transcription factors potentially regulating *Fbln1* identified ZNF384 as a shared regulatory element. WB analysis also demonstrated that the nuclear proteins interacting with the FB promoter include ZNF384 (Figure 4B). To confirm the specificity of this interaction, input samples from the DNA pull‐down assay have been included as loading controls. Depletion of ZNF384 resulted in a marked reduction in Fbln1 expression at both transcriptional and translational levels in cultured cells (Figure 4C–F). ChIP‐qPCR with a ZNF384‐specific antibody was performed to identify its genomic binding sites. Both ChIP‐seq profile (Figure 4G) and ChIP‐qPCR results demonstrated a direct association of ZNF384 with the promoter region of the *Fbln1* gene, as evidenced by a significant enrichment peak compared to the IgG control (Figure 4H). According to the JASPAR database prediction results, ZNF384 exhibits binding interactions with multiple regions of the *Fbln1* promoter, with the highest binding affinity observed at positions 1654–1655 bp. We designed *Fbln1* promoter mutants (1645 to 1655 bp) and conducted dual‐luciferase reporter assays in HEK‐293 T cells for further validation. The results demonstrated that ZNF384 significantly enhanced the luciferase activity of the wild‐type *Fbln1* 3’UTR (3’UTR‐wt) reporter construct, while its activating effect was attenuated in the mutant (Figure 4I). These findings demonstrate that ZNF384 can directly bind to the 1654–1655 nucleotide region of the *Fbln1* promoter and functionally activate its transcriptional activity.

We subsequently knocked down ZNF384 followed by overexpressing Fbln1 to determine whether the effects of ZNF384 on ECM deposition were mediated through Fbln1. As shown by WB in Figure S5, knockdown of ZNF384 reduced VSMC senescence and collagen expression, which was reversed by subsequent Fbln1 overexpression.

### 3.5 Knockdown of Fbln1 Suppressed the Expression of the TGF‐β/Smad3 Signaling Pathway in Senescent VSMCs

**FIGURE 5:**
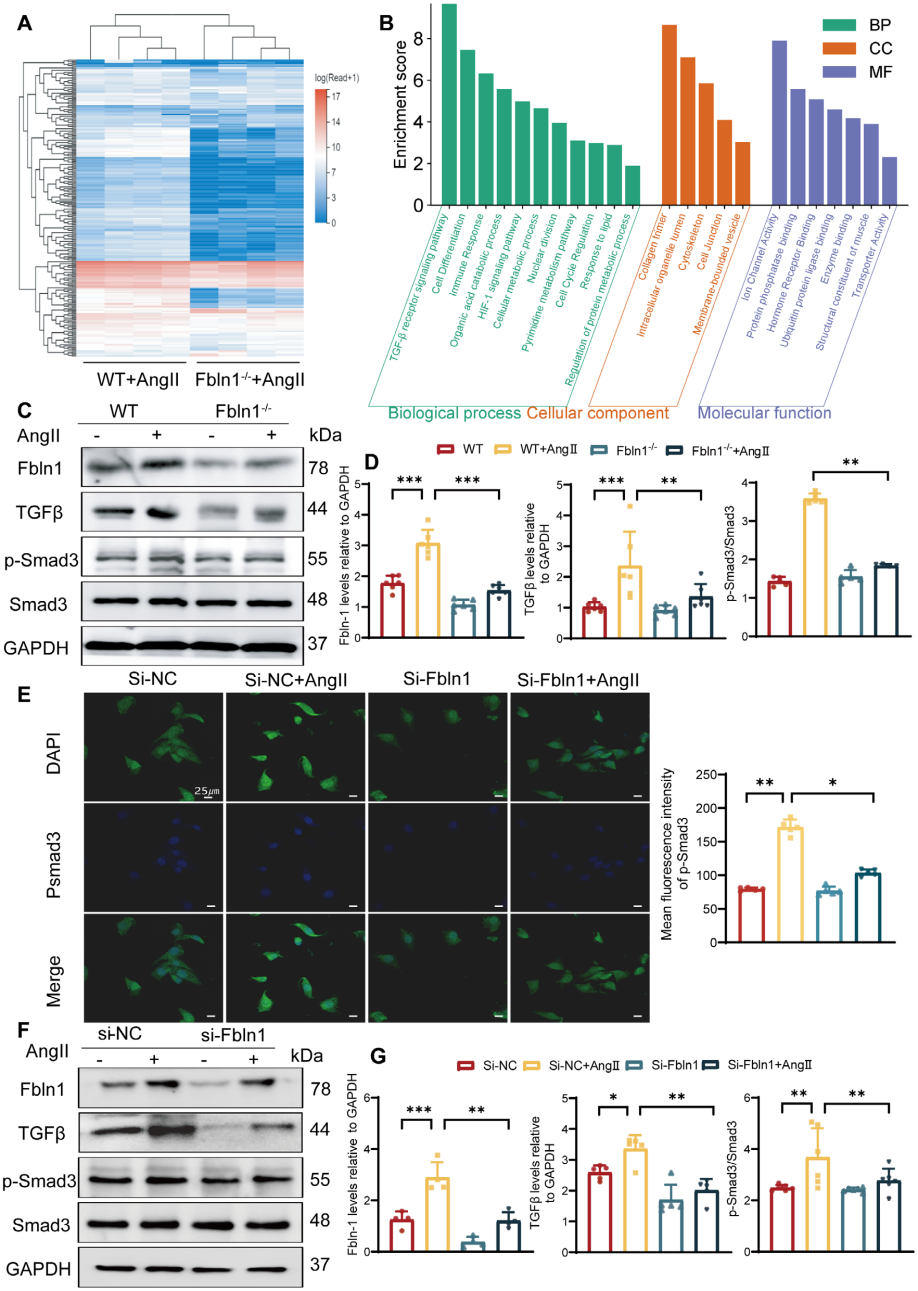
Knockdown of Fbln1 suppressed the expression of the TGF‐β/Smad3 signaling pathway. A–B: Bulk RNA‐sequence was performed in AngII‐induced vascular aging mice: Differential gene expression heatmap analysis (A) and Gene Ontology (GO) enrichment analysis of DEGs (B). C–D: Western Blot analysis was performed to detect the expression levels of Fbln1, TGF‐β, p‐Smad3/Smad3 in AngII‐treated and PBS control mice. E: Immunofluorescence staining showed the expression changes of p‐Smad3 after knocking down Fbln1 (scale = 50 μm). F–G: Western Blot analysis was performed to detect the expression levels of Fbln1, TGF‐β, p‐Smad3 and Smad3 in primary VSMCs after knocking down Fbln1. *n* ≥ 5, *p < 0.05, **p < 0.01, ***p < 0.001.

To delineate the downstream molecular mechanisms governing Fbln1‐mediated vascular stiffening, we conducted comparative transcriptomic profiling using bulk RNA‐sequencing (RNA‐seq) to delineate differential gene expression in VSMCs between Fbln1^−/−^ and WT mice following AngII induction. After homogenization analysis of all sample data in different groups, transcripts per million (TPM) are used to represent the relative abundance of a transcript among a population of sequenced transcripts. The filter parameter we set is that the log2 fold change (log2|FC|) > 1, false discovery rate (FDR) < 0.05. All differentially expressed genes (DEGs) were presented in a clustering heat map (Figure 5A). Gene Ontology (GO) clustering analysis of DEGs demonstrated significant enrichment of the TGF‐β signaling pathway within the biological process subcategory (Figure 5B). To investigate whether Fbln1 exerts its effects through the TGF‐β/Smad3 signaling pathway, we employed WB analysis to examine the influence of Fbln1 modulation on the activation of the TGF‐β/Smad3 signaling pathway in Fbln1^−/−^ mice. The results revealed that knockdown of Fbln1 led to a decrease in the expression of TGF‐β and p‐Smad3, while the expression of Smad3 remained largely unaffected (Figure 5C–D). Concomitant with Fbln1 deletion in VSMCs, both IF staining (for p‐Smad3) and WB analysis revealed profound inhibition of TGF‐β pathway activity (Figure 5E–G).

### 3.6 Inhibition of the TGF‐β/smad3 Signaling Pathway Relieves the Facilitating Effect of Fbln1 on VSMCs

**FIGURE 6:**
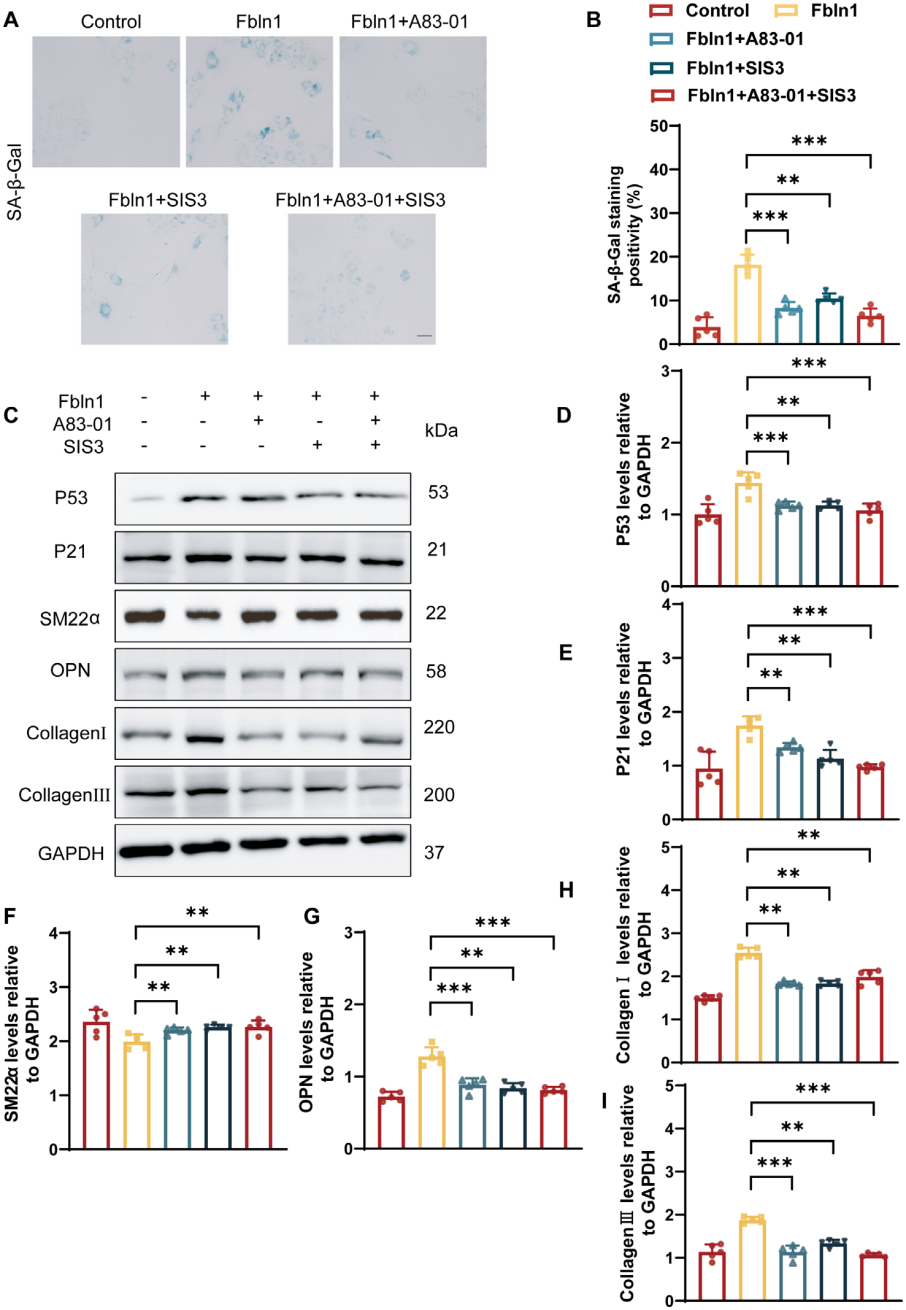
Inhibition of the TGF‐β/smad3 signaling pathway relieves the facilitating effect of Fbln1 on VSMCs. After treatment with recombinant Fibulin‐1 protein, the TGF‐β/Smad3 signaling pathway in VSMCs was blocked by adding the TGF‐β receptor inhibitor A83‐01 and the Smad3 phosphorylation inhibitor SIS3. A–B: Detection of VSMC senescence by senescence‐associated β‐galactosidase (SA‐β‐gal) staining. C–I: Western Blot was used to detect the expression levels of p53, p21, SM22α, OPN, collagenI and collagenIII. *n* ≥ 5, *p < 0.05, **p < 0.01, ***p < 0.001.

Previous experiments have demonstrated that Fbln1 can regulate the TGF‐β/Smad3 signaling pathway. In order to further verify whether Fbln1 participates in the regulation of vascular stiffness through similar circuitry, this experiment employed recombinant Fbln1 protein intervention in VSMCs, with the addition of 0.5 μM TGF‐β receptor inhibitor A83‐01 and 10 μM Smad3 phosphorylation inhibitor SIS3 to block the TGF‐β/Smad3 signaling pathway. Senescence‐associated β‐galactosidase staining demonstrated that recombinant Fbln1 accelerated VSMC senescence, which was effectively counteracted by inhibition of the TGF‐β/Smad3 signaling pathway through A83‐01 and SIS3 (Figure 6A,B). Consistent results were obtained through WB analysis (Figure 6C–E).

Additionally, blocking the TGF‐β/Smad3 signaling pathway improved the decreased expression of the contractile phenotypic marker αSMA, as evidenced by WB (Figure 6C,F). The recombinant Fbln1 protein‐induced upregulation of OPN, collagen I, and collagen III expression was significantly suppressed through pharmacological inhibition of the TGF‐β/Smad3 signaling pathway using A83‐01 and SIS3 (Figure 6C,G–I). Collectively, the pro‐senescent and collagen‐depositing effects of Fbln1 on VSMCs are mechanistically dependent on activation of the TGF‐β/Smad3 signaling axis.

## 4 Discussion

In this study, we successfully generated Fbln1 knockout mice and established vascular stiffness models via both natural aging and chronic AngII infusion to investigate the functional role of Fbln1 in pathological vascular remodeling. Experimental results demonstrated that Fbln1 downregulation significantly ameliorated vascular stiffness. Functionally, it reduced pulse wave velocity (PWV) and mean arterial pressure in mice. At the cellular and tissue levels, it enhanced VSMC relaxation capacity, attenuated ROS accumulation, improved collagen deposition, and restored elastic fiber arrangement. Mechanistically, our findings demonstrate that ZNF384 enhances Fbln1 transcription by binding to its promoter region, and Fbln1 subsequently promotes VSMC senescence and collagen deposition through a TGF‐β/Smad3 signaling‐dependent pathway.

Morphologically, vessels with increased stiffness exhibit elevated collagen fiber deposition accompanied by disorganized architecture and increased density of elastic fibers [23, 24]. Functionally, stiffened vascular is characterized by blunted responsiveness of VSMCs to vasodilators, heightened sensitivity to vasoconstrictors, and impaired angiogenic capacity [25]. Vascular stiffness also enhances susceptibility to hypertension and atherosclerosis. Elevated levels of Fbln1 are observed in the plasma of a familial cluster with vascular stiffness, suggesting an association between Fbln1 and the pathological increase in arterial stiffness. While hypertension is a known risk factor for vascular stiffness, the familial arteriosclerosis phenotype in our cohort appears to represent a distinct and more severe condition, independent of blood pressure elevation alone. Our study demonstrates that Fbln1 regulates vascular stiffness through dual mechanisms: modulating VSMC behavior and promoting collagen deposition. VSMCs are primarily located within the middle layer of blood vessel walls, and they participate in various physiological and pathological changes and are of great importance in maintaining the structural and functional integrity of the vessel wall [26, 27]. The phenotypic switching of SMCs plays a pivotal role in vascular remodeling and the pathogenesis of various vascular diseases. The VSMCs from a contractile to a synthetic phenotype is also recognized as a hallmark manifestation of cellular senescence [28]. Under normal physiological conditions, SMCs predominantly display a contractile phenotype, with their main function being the preservation of blood vessel diameter and contractile function [29]. However, in the course of vascular stiffness development during the process of vascular aging, there is an increase in the expression of inflammatory factors and disturbances in calcium and phosphate metabolism, leading to a series of pathological and physiological changes [30]. These age‐related alterations can induce various secondary responses within VSMCs, promoting their transition from contractile to synthetic (non‐contractile) phenotypes. This leads to changes in vascular structure and function, contributing to the process of vascular stiffness [31, 32, 33]. This study reveals that vascular stiffness exhibits an age‐dependent increase, and aging and Ang II induction synergistically trigger VSMC senescence, accompanied by a shift toward a synthetic phenotype. Fbln1 knockdown markedly attenuated the expression of aging‐related molecules, such as p53 and p21. Aging triggers increased endogenous ROS production in VSMCs, which in turn impairs mitochondrial function, establishing a self‐amplifying vicious cycle. This detrimental cycle is mitigated by Fbln1 deficiency, which reduces ROS accumulation [34]. Through the assessment of protein markers, including SM22α, αSMA and OPN, it was observed that knockdown of Fbln1 improves the phenotypic transition of SMCs, suggesting that Fbln1 may participate in the process of vascular aging by regulating SMC phenotypic transformation. We further established that the loss of the VSMC contractile phenotype leads to diminished responsiveness to vasodilators and compromised relaxation. Importantly, Fbln1 knockdown effectively rescued this deficit in endothelium‐independent vasodilation. Furthermore, Fbln1 downregulation improved collagen deposition and elastic fiber arrangement in vascular tissues.

The investigation of the molecular mechanisms regulating Fbln1 expression aims to elucidate its transcriptional control network, thereby providing a foundational rationale for developing targeted therapeutic strategies. A DNA pull‐down assay was performed by incubating the *Fbln1* promoter region with nuclear protein extracts, followed by mass spectrometry‐based proteomic analysis of the captured protein complexes. The proteins pulled down in this assay are likely to include transcription factors (TFs) and transcriptional co‐regulators, which typically bind to cis‐regulatory elements within gene promoter regions to orchestrate transcriptional activation or repression. Combined with TF binding site prediction, we have identified ZNF384, which directly binds to the *Fbln1* promoter, as the candidate transcriptional regulator of its expression. Knockdown of ZNF384 led to decreased Fbln1 expression. Consistently, ChIP experiments confirmed a significant binding peak of ZNF384 within the *Fbln1* promoter region. Further dual‐luciferase assays demonstrated that this binding is functional, as ZNF384 directly promotes *Fbln1* transcription via a specific site located at the 1654–1655 bp region of its promoter. While ZNF384 has been implicated in ECM regulation, its specific role in cardiovascular diseases remains poorly defined. This study demonstrated that while knockdown of ZNF384 ameliorates the VSMC senescence phenotype and reduces collagen deposition, overexpression of Fbln1 was able to rescue the phenotypes induced by ZNF384 deficiency. Our study provides emerging evidence supporting ZNF384 as a potential upstream regulator in cardiovascular pathophysiology, potentially functioning through modulation of Fbln1 expression.

To further delineate the downstream signaling networks regulated by Fbln1, we conducted RNA‐seq to systematically profile transcriptomic changes associated with Fbln1. GO analysis revealed that DEGs were significantly enriched in the TGF‐β signaling pathway. Furthermore, both in vitro and in vivo experiments demonstrated suppression of TGF‐β/Smad3 signaling following Fbln1 depletion. TGF‐β, functioning as a multifunctional cell cytokine, participates in regulating the phenotypic transformation of SMCs and holds a significant role [35]. Smad3 is of essential importance in the TGF‐β/Smad signaling pathway, which is the classic pathway of TGF‐β. This pathway actively participates in various cardiovascular diseases, such as atherosclerosis, as well as in regulating multiple biological activities of SMCs and inflammatory cells [6, 8, 15, 16, 35, 36, 37, 38, 39, 40, 41, 42, 43]. We found that, compared to the control group, phosphorylation of Smad3 increased in aging SMCs. Following canonical activation of the TGF‐β pathway, p‐Smad3 then translocates into the cell nucleus to exert its function. As such, this experiment further revealed increased expression and nuclear translocation of p‐Smad3 through IF staining [43]. This connection is consistent with previous research in the context of pulmonary fibrosis, where Fbln1 was found to modulate collagen secretion in lung fibroblasts through the TGF‐β/Smad3 signaling pathway [16].

To investigate the involvement of the TGF‐β/Smad3 pathway in Fbln1's role in VSMC aging, Fbln1 knockdown experiments were conducted. These experiments revealed that reducing Fbln1 expression led to diminished TGF‐β levels and lowered Smad3 phosphorylation levels, suggesting that Fbln1 could indeed activate the TGF‐β/Smad3 pathway in senescent smooth muscle cells.

Further studies are warranted to determine whether Fbln1 mechanistically depends on the TGF‐β/Smad3 signaling pathway to regulate vascular stiffening. This was examined by utilizing inhibitors A83‐01, a TGF‐β receptor inhibitor, and SIS3, a Smad3 phosphorylation inhibitor, to block the TGF‐β/Smad3 pathway. The results demonstrated that these inhibitors curbed the aging‐promoting impact of exogenous recombinant Fbln1 protein on VSMCs. Evaluation of SMC phenotypic transition markers indicated that obstructing TGF‐β/Smad3 pathway activation could mitigate Fbln1's role in inducing phenotypic transition in these cells. Similarly, Fbln1 also depends on the activation of TGF‐β signaling to promote collagen expression.

In summary, this study identified elevated plasma Fbln1 levels in a family cohort with hereditary vascular stiffening. Knockdown of Fbln1 may reduce vascular stiffness by attenuating VSMC senescence and collagen deposition. A potential mechanistic axis involves ZNF384‐driven transcriptional activation of Fbln1, which subsequently stimulates the TGF‐β/Smad3 signaling pathway to promote vascular stiffening via dysregulated vascular remodeling. These findings provide novel theoretical support for the potential of Fbln1 as a marker of vascular stiffness and hold significant implications for enhancing the assessment framework of vascular stiffness and advancing early prevention and intervention strategies.

## Author Contributions

Dan Yan and Tianyi Ji contributed equally to this work and should be considered joint first authors. Lei Ran and Cuntai Zhang were mainly responsible for project design and provided funding sources. Dan Yan, Tianyi Ji, Xiaolu Liang, Yi Huang, and Mandi Luo contributed to performing mainly animal and cell experiments. Pengcheng Luo and Zhen Yang assisted in data analysis. Le Zhang, Yongping Bai, and Tao Li contributed to the manuscript draft. The authors did not use generative AI or AI‐assisted technologies in the development of this manuscript.

## Funding

This work was supported by the Hubei Province Innovation Project (Grant No. 2024BCB045) and the National Natural Science Foundation of China (Grant No. 82571792).

## Conflicts of Interest

The authors declare no conflicts of interest.

## Supporting information


**Figure S1:** A: Pulse wave velocity (PWV) in young and aged mice. B–D: Western blot results showed the expression levels of p53, p21 in vascular tissues of naturally aged mice. E: Immunofluorescence analysis of vascular tissues in young and elderly cohorts. L, lumen; M, tunica media; A, tunica adventitia. F–G: Genotyping of WT and Fbln1^−/−^ mice. H: Western blot results showed the expression levels of Fbln1 in WT and Fbln1^−/−^ mice. *Significant differences. *n* ≥ 5, *p < 0.05, **p < 0.01, ***p < 0.001.
**Figure S2:** Knockdown of Fbln1 in vivo remediated VSMC phenotypic transition and collagen deposition. A: Immunohistochemical (IHC) staining of Fbln1 expression in WT and Fbln1^−/−^ mice. wild‐type mice: WT, Fbln1 knockout mice: Fbln1^−/−^. B: Comparison of PWV in WT and Fbln1^−/−^ mice, stratified by sex (male and female). C–D: HE staining showed the wall thickness in normal WT and Fbln1^−/−^ mice (scale = 100 μm). Assessment of collagen deposition in vascular tissues by Masson's trichrome staining in WT and Fbln1^−/−^ mice (scale = 50 μm). E: Measurement of smooth muscle cell‐mediated relaxation capacity in WT and Fbln1^−/−^ mice. F–G: Detection of reactive oxygen species (ROS) in vascular tissues (scale = 40 μm). *Significant differences. *n* ≥ 5, *p < 0.05, **p < 0.01, ***p < 0.001.
**Figure S3:** Knockdown of Fbln1 in vivo remediated VSMC phenotypic transition and collagen deposition. A–G: Western blot results showed that Fbln1 knockdown could modulate the expression of αSMA, SM22α, OPN, collagenI and collagenIII in WT and Fbln1^−/−^ mice. wild‐type mice: WT, Fbln1 knockout mice: Fbln1^−/−^. H: Measurement of smooth muscle cell‐mediated relaxation capacity in WT and Fbln1^−/−^ mice. I: Detection of reactive oxygen species (ROS) in vascular tissues (scale = 40 μm). *Significant differences. *n* ≥ 5, *p < 0.05, **p < 0.01, ***p < 0.001.
**Figure S4:** Knockdown of Fbln1 ameliorated VSMC aging and phenotypic transition. A–C: Inhibition of Fbln1 expression in VSMCs via siRNA transfection: siRNA resulted in decreased Fbln1 both mRNA and protein levels. D–L: 10^−7^ mol/L AngII stimulated smooth muscle cells for 72 h, inducing VSMC senescence. D–E: Detection of VSMC senescence by senescence‐associated β‐galactosidase (SA‐β‐gal) staining. F–H: Western blot results showed that Fbln1 knockdown could reduce the expression of aging related proteins P53 and P21 induced by AngII in VSMCs. I–L: Western Blot analysis was performed to detect the expression levels of phenotypic transformation markers αSMA, SM22α, OPN, of VSMCs after Fbln1 down‐tapping. *n* ≥ 4, *p < 0.05, **p < 0.01, ***p < 0.001.
**Figure S5:** Overexpression of Fbln1 rescues the phenotype induced by ZNF384 deficiency. VSMCs treated with AngII were subjected to either knockdown of ZNF384 or overexpression of Fbln1. A: Western Blot analysis was performed to detect the expression levels of p53, p21, collagenI and collagenIII. *n* ≥ 4, *p < 0.05, **p < 0.01, ***p < 0.001.

**Table S1:** Vascular stiffness pedigree information.
**Table S2:** Carotid surgery patient information.

## Data Availability

The data that support the findings of this study are available from the corresponding author upon reasonable request.
