# Supplementary material for: ZNF384‐Driven Fibulin‐1 Exacerbates Vascular Stiffness via TGF‐β/Smad3‐Mediated Senescence and Fibrosis
Source: FASEB J. 2026 Mar 12;40(6):e71599. doi: 10.1096/fj.202501262RR (PMC12980562; doi:10.1096/fj.202501262RR)

Supplemantary Figure 1

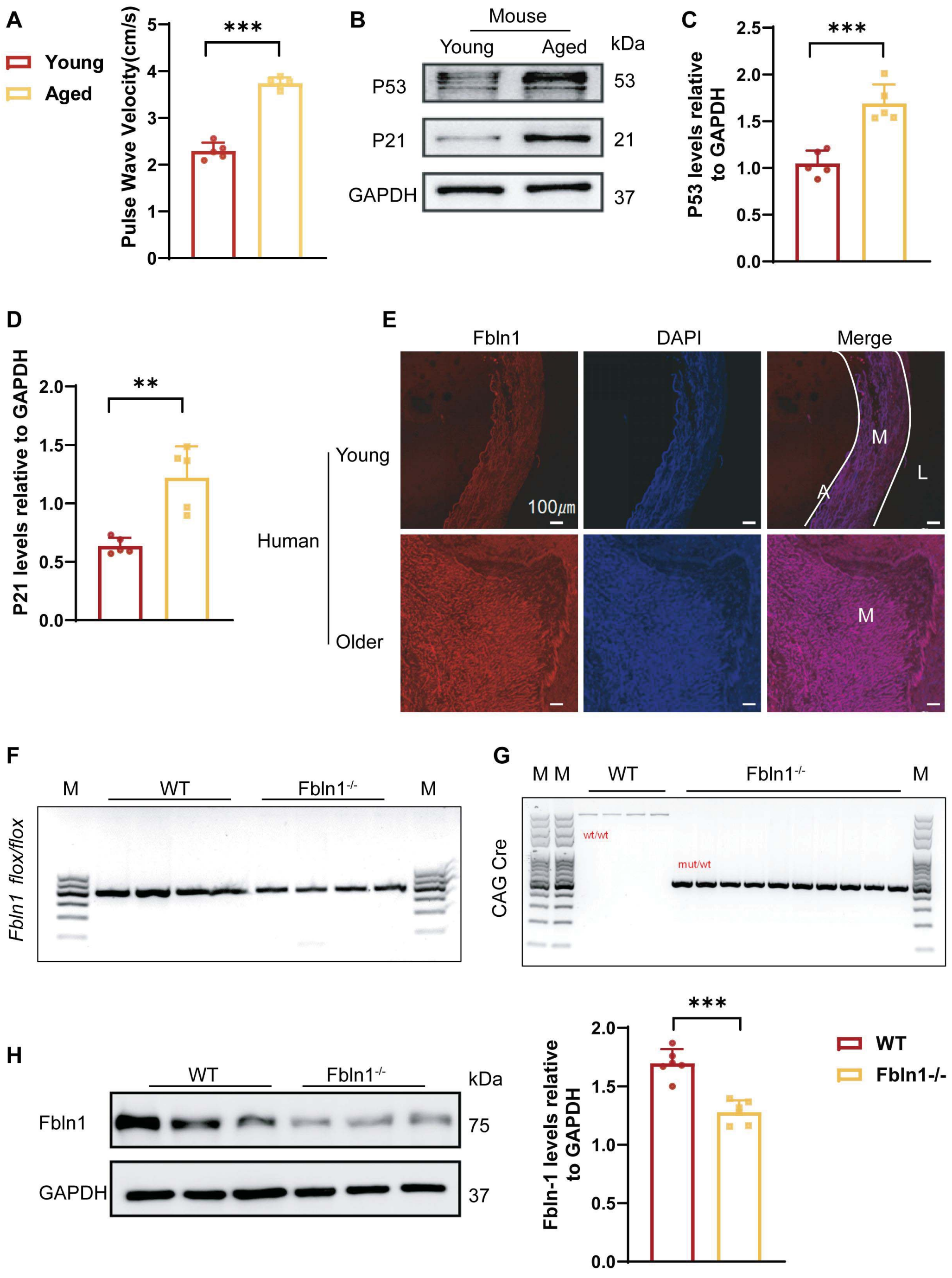

Supplemantary Figure 2

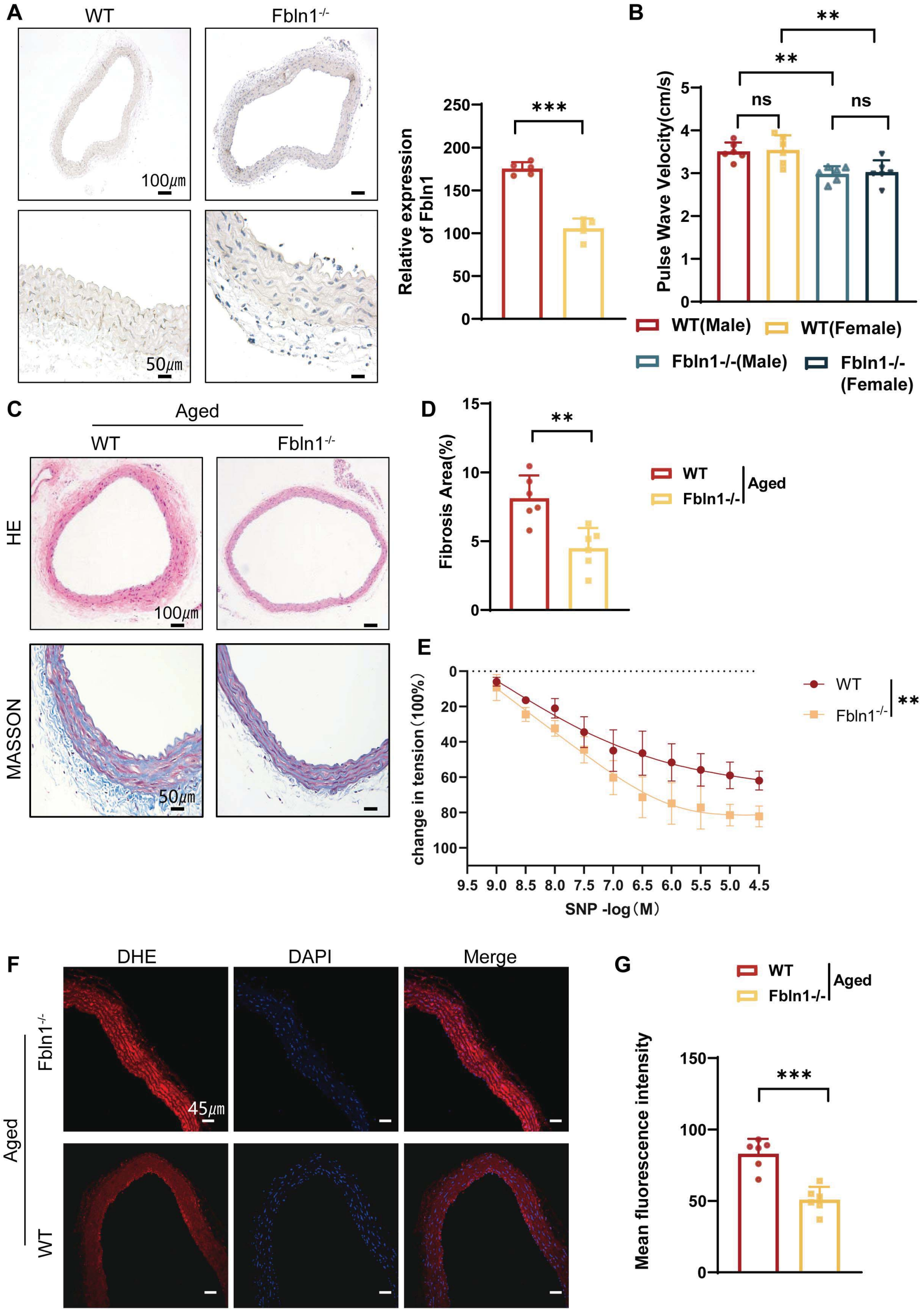

Supplemantary Figure 3

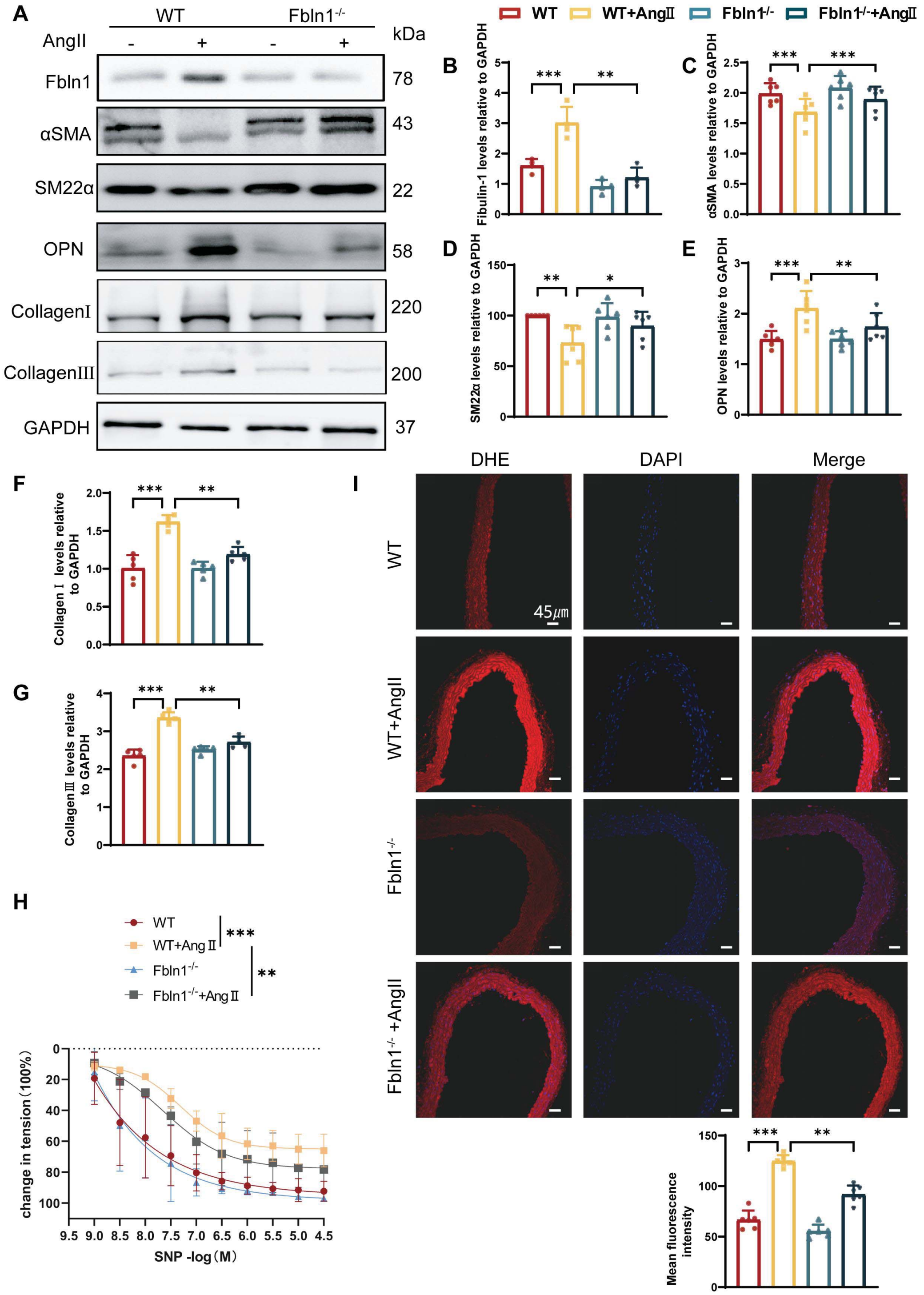

Supplemantary Figure 4

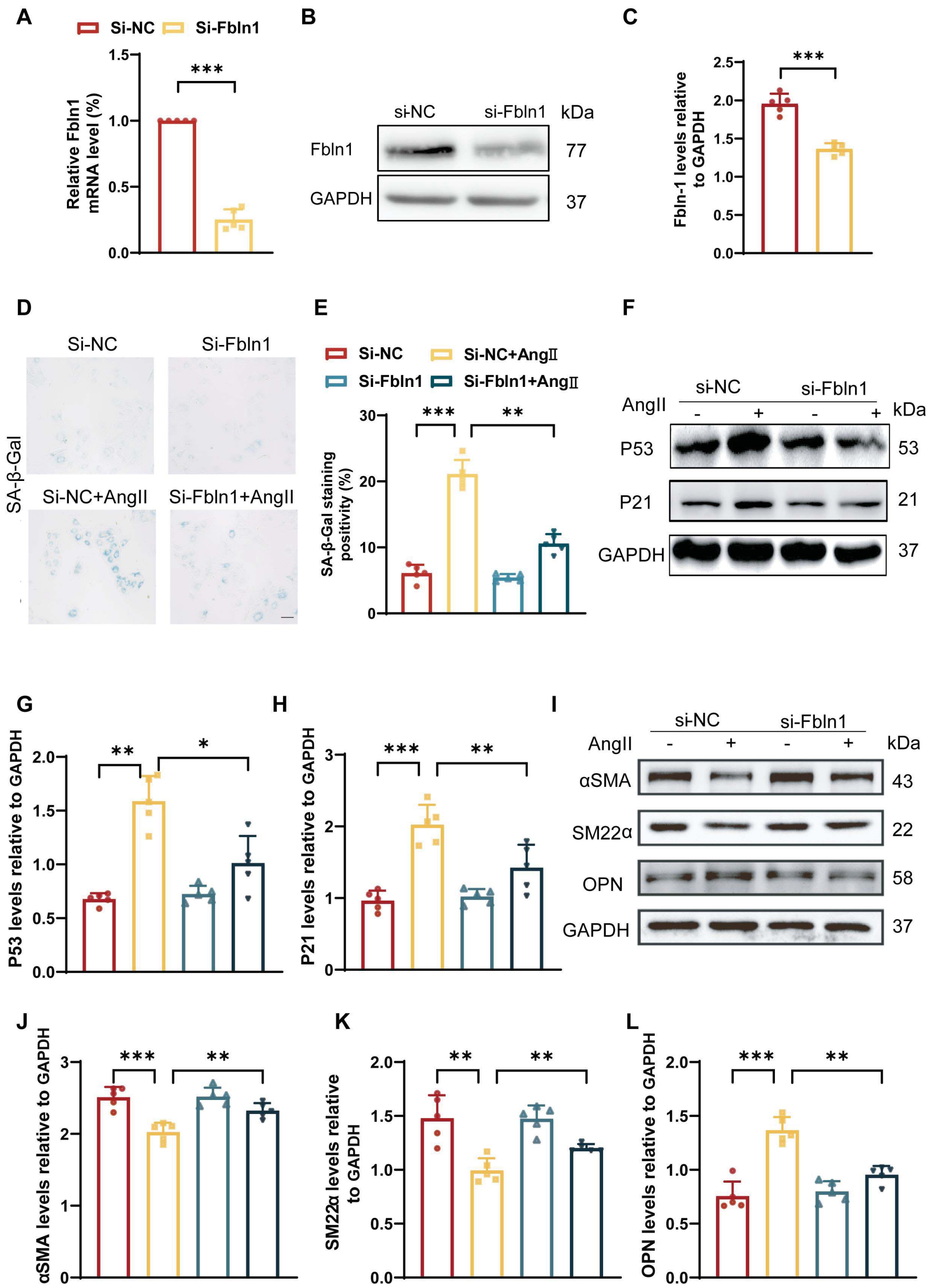

Supplemantary Figure 5

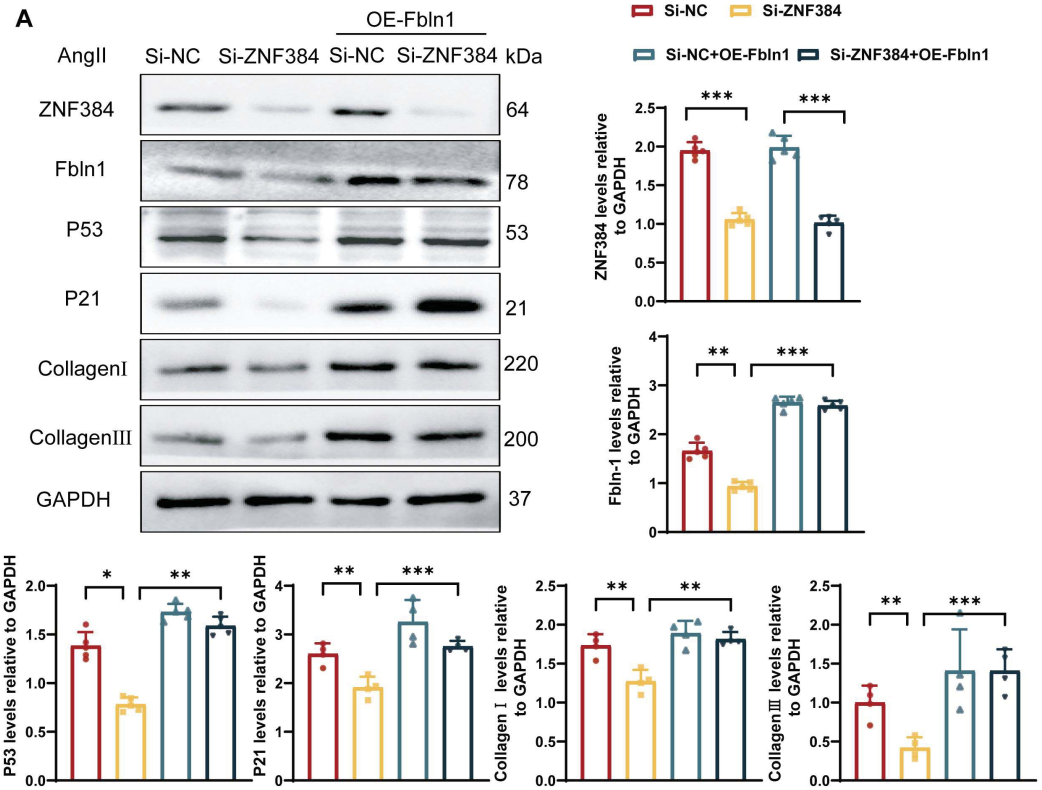

Supplement: Supplementary file 1 — Figure S1: A: Pulse wave velocity (PWV) in young and aged mice. B–D: Western blot results showed the expression levels of p53, p21 in vascular tissues of naturally aged mice. E: Immunofluorescence analysis of vascular tissues in young and elderly cohorts. L, lumen; M, tunica media; A, tunica adventitia. F–G: Genotyping of WT and Fbln1−/− mice. H: Western blot results showed the expression levels of Fbln1 in WT and Fbln1−/− mice. *Significant differences. n ≥ 5, *p < 0.05, **p < 0.01, ***p < 0.001. Figure S2: Knockdown of Fbln1 in vivo remediated VSMC phenotypic transition and collagen deposition. A: Immunohistochemical (IHC) staining of Fbln1 expression in WT and Fbln1−/− mice. wild‐type mice: WT, Fbln1 knockout mice: Fbln1−/−. B: Comparison of PWV in WT and Fbln1−/− mice, stratified by sex (male and female). C–D: HE staining showed the wall thickness in normal WT and Fbln1−/− mice (scale = 100 μm). Assessment of collagen deposition in vascular tissues by Masson's trichrome staining in WT and Fbln1−/− mice (scale = 50 μm). E: Measurement of smooth muscle cell‐mediated relaxation capacity in WT and Fbln1−/− mice. F–G: Detection of reactive oxygen species (ROS) in vascular tissues (scale = 40 μm). *Significant differences. n ≥ 5, *p < 0.05, **p < 0.01, ***p < 0.001. Figure S3: Knockdown of Fbln1 in vivo remediated VSMC phenotypic transition and collagen deposition. A–G: Western blot results showed that Fbln1 knockdown could modulate the expression of αSMA, SM22α, OPN, collagenI and collagenIII in WT and Fbln1−/− mice. wild‐type mice: WT, Fbln1 knockout mice: Fbln1−/−. H: Measurement of smooth muscle cell‐mediated relaxation capacity in WT and Fbln1−/− mice. I: Detection of reactive oxygen species (ROS) in vascular tissues (scale = 40 μm). *Significant differences. n ≥ 5, *p < 0.05, **p < 0.01, ***p < 0.001. Figure S4: Knockdown of Fbln1 ameliorated VSMC aging and phenotypic transition. A–C: Inhibition of Fbln1 expression in VSMCs via siRNA transfection: siRNA resulted [file FSB2-40-e71599-s001.pdf]
