# Supplementary material for: ZNF384‐Driven Fibulin‐1 Exacerbates Vascular Stiffness via TGF‐β/Smad3‐Mediated Senescence and Fibrosis
Source: FASEB J. 2026 Mar 12;40(6):e71599. doi: 10.1096/fj.202501262RR (PMC12980562; doi:10.1096/fj.202501262RR)
Supplement: Supplementary file 2 — Table S1: Vascular stiffness pedigree information. Table S2: Carotid surgery patient information. [file FSB2-40-e71599-s002.docx]

Table S1. Vascular stiffness pedigree information.

| Nubmer | Age | Gender | Blood pressure (mmHg) |
| --- | --- | --- | --- |
| Vascular Stiffness Pedigree 1 | 78 | Male | 141/89 |
| Vascular Stiffness Pedigree 2 | 72 | Male | 136/78 |
| Vascular Stiffness Pedigree 3 | 62 | Male | 142/72 |
| Vascular Stiffness Pedigree 4 | 48 | Female | 135/81 |
| Vascular Stiffness Pedigree 5 | 51 | Male | 150/104 |
| Vascular Stiffness Pedigree 6 | 48 | Male | 124/70 |
| Vascular Stiffness Pedigree 7 | 43 | Male | 130/75 |
| Family Control 1 | 58 | Female | 137/84 |
| Family Control 2 | 50 | Female | 144/92 |
| Family Control 3 | 52 | Female | 134/77 |
| Family Control 4 | 47 | Male | 146/82 |
| Family Control 5 | 48 | Male | 139/74 |
| Family Control 6 | 37 | Female | 125/68 |
| Family Control 7 | 22 | Female | 108/55 |

A familial cluster with pathologically increased arterial stiffness was identified through routine cardiovascular screening at Tongji Hospital. Affected individuals manifested accelerated arteriosclerosis, as confirmed by elevated pulse wave velocity (PWV ≥ 1400 cm/s), while unaffected family members served as internal controls (PWV within age-adjusted normal ranges). Inclusion criteria: (1) age ≥ 18 years; (2) availability of detailed medical history; (3) absence of secondary causes of arteriosclerosis (e.g., chronic kidney disease, diabetes mellitus). Exclusion criteria: (1) acute cardiovascular events within the preceding 6 months; (2) use of vasoactive medications (e.g., nitrates, calcium channel blockers). Peripheral blood samples were collected under fasting conditions.

Table S2. Carotid surgery patient information.

| Nubmer | Age | Gender | Blood pressure (mmHg) |
| --- | --- | --- | --- |
| Older1 | 61 | Male | 145/81 |
| Older2 | 72 | Male | 151/92 |
| Older3 | 66 | Male | 138/72 |
| Older4 | 63 | Female | 160/85 |
| Older5 | 69 | Male | 128/71 |
| Young1 | 34 | - | - |
| Young2 | 42 | - | - |
| Young3 | 31 | - | - |
| Young4 | 35 | - | - |
| Young5 | 29 | - | - |

Vascular tissues from elderly patients (n=5) were collected from the cardiovascular tissue repository under the Department of Cardiovascular Surgery at Tongji Hospital (2019–2021). Vascular tissues from young donors were obtained during organ procurement procedures for heart transplantation.
